# Validity and safety of ID-JPL934 in lower gastrointestinal symptom improvement

**DOI:** 10.1038/s41598-021-92007-3

**Published:** 2021-06-22

**Authors:** Cheol Min Shin, Yoon Jin Choi, Dong Ho Lee, Jin Seok Moon, Tae-Yoon Kim, Yoon-Keun Kim, Won-Hee Lee, Hyuk Yoon, Young Soo Park, Nayoung Kim

**Affiliations:** 1 Department of Internal Medicine, Seoul National University Bundang Hospital, 82, Gumi-ro 173, Beon-gil, Seongnam, Gyeonggido 13620 South Korea; 2 Research Laboratories, ILDONG Pharmaceutical Co., Ltd., Hwaseong, South Korea; 3 MD Healthcare Inc., Seoul, Republic of Korea

**Keywords:** Clinical microbiology, Microbiome, Gastrointestinal diseases, Drug development, Microbiology

## Abstract

The study evaluated the efficacy of ID-JPL934, a probiotic preparation containing *Lactobacillus johnsonii* IDCC 9203, *Lactobacillus plantarum* IDCC 3501 and *Bifidobacterium lactis* IDCC 4301, in relieving lower gastrointestinal symptoms. A total of 112 subjects with lower gastrointestinal symptoms were consecutively enrolled. They were randomized into either ID-JPL934 administration group or placebo group. Bristol stool form, stool frequency, and abnormal bowel movement symptoms were recorded at baseline and week 2, 6, and 8. Primary endpoint was improvement in overall symptoms at week 8. Fecal samples were collected to measure the probiotic levels in feces using quantitative polymerase chain reaction (qPCR), and to perform metagenomic analysis of microbiome originating from bacteria-derived extracellular vesicles and bacterial cells via 16S rDNA sequencing. Of the 112 subjects, 104 (54 in ID-JPL934 group and 50 in placebo group) completed the entire study protocol. A higher relief of overall symptoms was found in ID-JPL934 group than in placebo group (*p* = 0.016). Among lower gastrointestinal symptoms, abdominal pain and bloating scores were more decreased in ID-JPL934 group than in placebo group (*p* < 0.05). The fecal microbiome profiles of the two groups did not differ. However, the qPCR analysis showed significant increase in the levels of *Lactobacillus johnsonii* and *Bifidobacterium lactis* in feces post-treatment in ID-JPL934 group than in placebo group (*p* < 0.05 by repeated measure ANOVA). In conclusion, ID-JPL934 is effective in relieving lower gastrointestinal symptoms. Exposure to ID-JPL934 may increase the abundance of *Lactobacillus johnsonii* and *Bifidobacterium lactis* in the gut.

**Trial registration:** ClinicalTrials.gov number, NCT03395626.

## Introduction

Lower gastrointestinal symptoms generally require a visit to a physician. The diversity of clinical presentations and underlying etiologies limit the treatment options as no single dominant drug is effective in all cases. Despite the discovery of new pharmacological treatments, it is a challenge to identify drugs that can improve lower gastrointestinal symptoms without inducing adverse effects^1,2^.

As gut microbiota are important in health and disease, there is a growing body of evidence to suggest the therapeutic potential of probiotics in functional gastrointestinal disorders such as irritable bowel syndrome (IBS) and other conditions^3^. It has been suggested that probiotics can relieve various abdominal symptoms^4,5^. To date, the proposed mechanisms of action underlying the beneficial effects of probiotics include competitive exclusion of pathogenic microorganisms, inhibition of pathogen adhesion, production of anti-microbial substances, and modulation of the immune system^6,7^.

Whether multi-strain probiotic supplementation is superior to a single strain in ameliorating various gastrointestinal symptoms is controversial^8^. Nevertheless, ID-JPL934, which is a combination of *Lactobacillus johnsonii* IDCC 9203 (isolated from infant feces), *Lb. plantarum* IDCC 3501 (isolated from Kimchi), and *Bifidobacterium animalis* subspecies *lactis* IDCC 4301 (isolated from infant feces), has shown considerably higher levels of anti-oxidative and α-glucosidase inhibiting activity, as well as greater inhibition of nitric oxide synthesis than other candidate strains^9^. It substantially inhibited the release of inflammatory mediators such as tumor necrosis factor (TNF)-α, interleukin (IL)-6, and IL-1β by RAW 264.7 macrophages treated with lipopolysaccharide^9^. In a recent study, the probiotic strains improved the symptoms of dextran sodium sulfate-induced colitis in mice, in a model of ulcerative colitis^9^. However, the effectiveness of ID-JPL934 in alleviating abdominal symptoms has yet to be evaluated in human study.

Accordingly, the objective of this randomized, double-blind, placebo-controlled, parallel-group trial was to evaluate the validity and safety of ID-JPL934 in ameliorating lower gastrointestinal symptoms such as constipation, diarrhea, and abdominal pain.

## Results

### Characteristics of the study participants

Of the 112 study participants, 104 subjects (54 in the ID-JPL934 group and 50 in the placebo group) completed the entire study protocol without any serious adverse effect. There was no significant difference in baseline characteristics between the two groups except for flatulence (Table 1). The two groups showed no significant differences in dietary profiles not only at the baseline but also at week 8 (Supplementary Table S1). No adverse reactions were reported by the study participants related to either ID-JPL934 or placebo.

**Table 1:** Baseline characteristics of study subjects.

	ID-JPL934 group	Placebo group	*P*-value
	(n = 56)	(n = 56)	
Age	38.9 ± 12.2	37.3 ± 1.7	0.467
Male, n (%)	14 (25.0)	21 (37.5)	0.259
Smoker, n (%)	7 (12.5)	5 (9.1)	0.795
Drinker, n (%)	30 (53.6)	38 (69.1)	0.178
Body mass index (kg/m^2^)	27.9 ± 4.1	27.4 ± 4.5	0.553
Stool form (BSFS)	4.14 ± 1.58	4.00 ± 1.75	0.653
**Abnormal bowel movement symptoms**			
Stool frequency/day	1.47 ± 1.04	1.52 ± 1.12	0.808
Abdominal pain^a^	2.78 ± 1.64	2.96 ± 1.71	0.576
Abdominal discomfort^a^	3.03 ± 1.54	3.03 ± 1.75	0.955
Constipation score^a^	2.08 ± 1.79	2.55 ± 2.35	0.243
Diarrhea score^a^	2.78 ± 2.64	2.85 ± 3.02	0.894
Abdominal bloating^a^	3.30 ± 2.13	3.51 ± 2.68	0.641
Flatulence^a^	3.05 ± 2.11	4.00 ± 2.55	**0.035**
**Dietary intakes**			
Calories (kcal/day)	1,671.8 ± 448.7	1,585.2 ± 388.8	0.280
Carbohydrate (g/day)	231.9 ± 65.9	229.0 ± 63.4	0.815
Fat (g/day)	50.2 ± 18.7	44.1 ± 14.1	0.055
Protein (g/day)	64.5 ± 20.8	60.9 ± 16.4	0.826
Dietary fiber (g/day)	17.4 ± 5.9	17.1 ± 6.3	0.316
Folate (μg/day)	334.1 ± 108.8	329.3 ± 103.1	0.810
Calcium (mg/day)	377.5 ± 155.7	345.3 ± 155.8	0.279

Data presented as mean ± SD. *P*-values were calculated by either χ^2^ test (categorical variables) or Student's t-test (continuous variables).

^a^Visual analog scale (0–10). BMI, body mass index; BSFS, Bristol Stool Form Scale. Bold style indicates statistical significance.

### Improvement in overall symptoms at week 8

**Figure 1:**
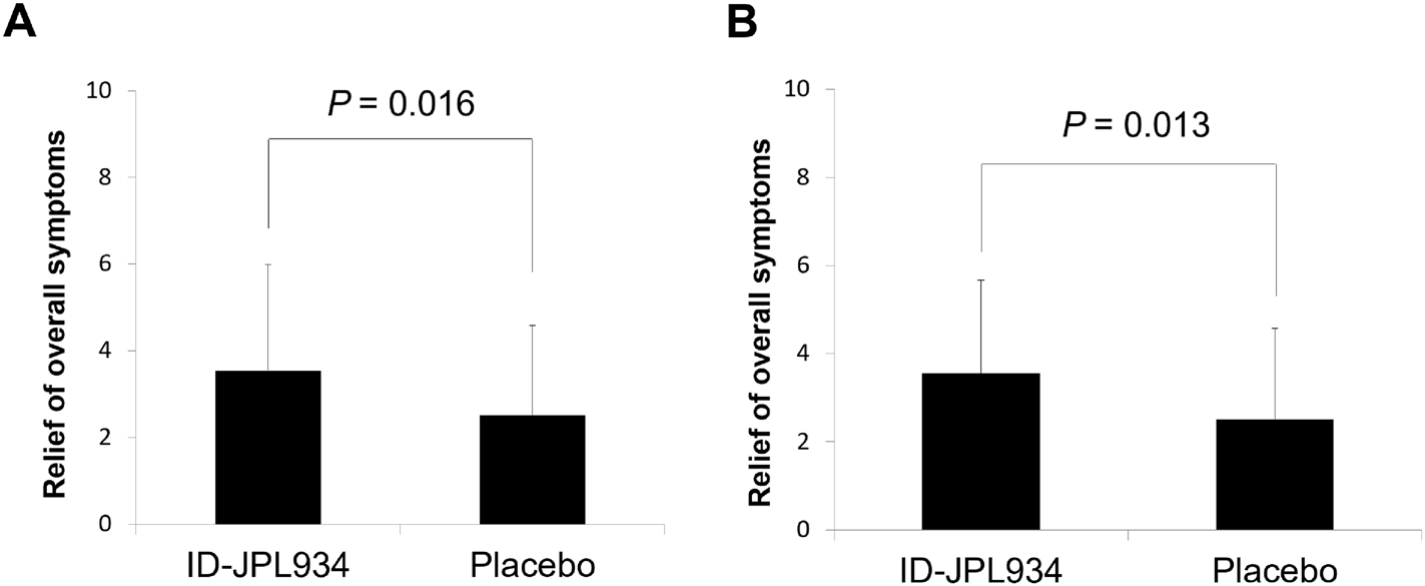
Relief of overall symptoms in the ID-JPL934 group *vs.* placebo group after 8-week treatment. (**A**) Intention-to-treat analysis (n = 112). (**B**) Per protocol analysis (n = 104). A significantly higher relief of overall symptoms was observed in the ID-JPL934 than in the placebo group. *P*-values were calculated using Student’s t-test.

A significantly higher relief of overall lower abdominal symptoms was observed in the ID-JPL934 group than in the placebo group in the intention-to-treat analysis (*p* = 0.016, Fig. 1). This result was also significant in per-protocol analysis (*p* = 0.012).

### Changes in lower abdominal symptoms during the study period

Changes of BSFS, stool frequency, and the 10-point VAS scores in abnormal bowel movement symptoms at weeks 0, 2, 6, and 8 are summarized in Supplementary Figure S1. Abdominal pain, abdominal discomfort, bloating, and flatulence scores were significantly lower in the ID-JPL934 group than in the control group (*p* < 0.05). A comparison of the two groups for changes in stool form, stool frequency, and abnormal bowel movement symptoms from baseline to week 8 revealed a higher decrease in abdominal pain and bloating in the ID-JPL934 group than in the placebo group, suggesting significant differences between the two groups (*p* < 0.05 by Student’s t-test, Table 2).

**Table 2:** Comparison of changes in stool form, frequency of bowel movements per day, and abnormal bowel movement symptoms from baseline to week 8 between the 2 groups.

	ID-JPL934 (n = 54)	Placebo (n = 50)	*P*-value
	Mean ± SD	Mean ± SD	
Stool form (BSFS)	0.06 ± 1.73	− 0.08 ± 1.77	0.694
Stool frequency/day	0.39 ± 2.08	0.31 ± 1.36	0.826
Abdominal pain^a^	− 1.83 ± 1.96	− 1.06 ± 1.65	**0.048**
Abdominal discomfort^a^	− 1.90 ± 1.82	− 1.20 ± 1.80	0.053
Constipation score^a^	− 1.24 ± 2.24	− 0.74 ± 2.36	0.275
Diarrhea score^a^	− 1.83 ± 2.50	− 1.24 ± 2.66	0.241
Abdominal bloating^a^	− 2.03 ± 2.76	− 0.84 ± 2.33	**0.020**
Flatulence^a^	− 1.59 ± 1.89	− 1.44 ± 1.94	0.685

Per protocol analysis. *P*-values were calculated using Student's t-test. Bold style denotes statistical significance.

^a^Visual analog scale (0–10). BSFS, Bristol Stool Form Scale; SD, standard deviation.

### Metagenomic analyses of participants’ fecal samples

To evaluate whether supplementation of the multi-strain probiotics (ID-JPL934) changed the gut microbiota profile, the fecal samples from the two groups were analyzed before and after treatment using Illumina MiSeq platform targeting 16S rDNA following the isolation of extracellular veiscles (EVs) from feces.

**Figure 2:**
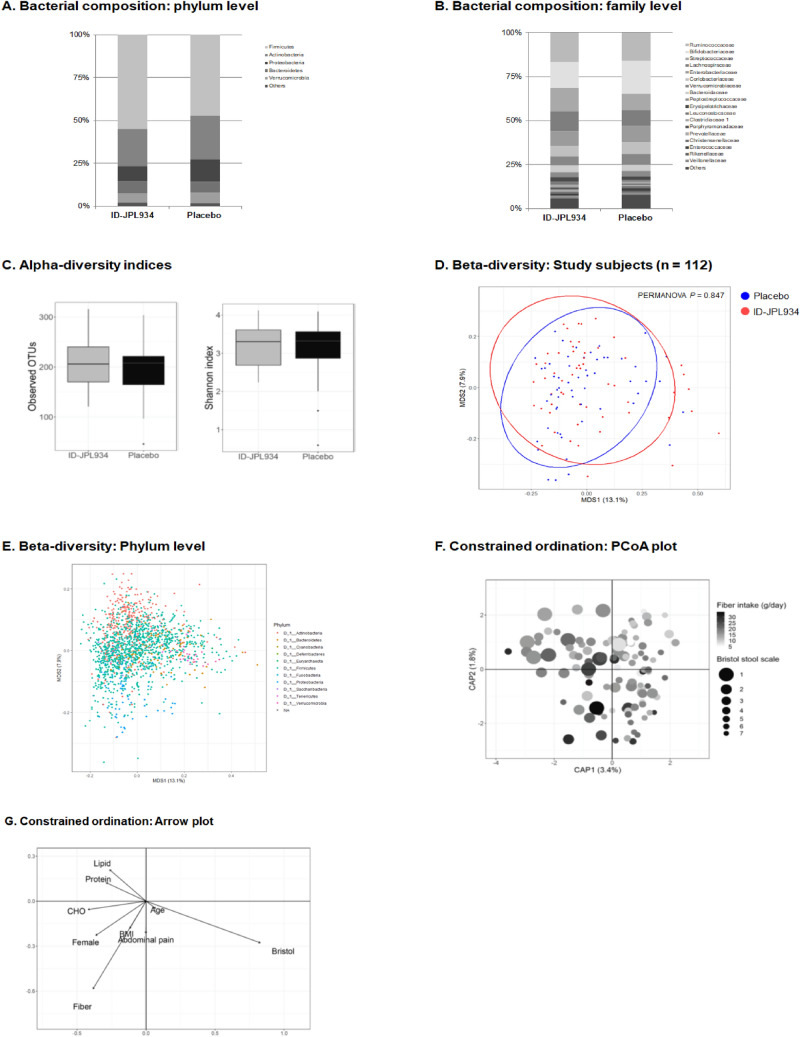
Summary of the metagenomic analysis of the microbiome originating from bacterial cells at baseline. The placebo and the ID-JPL934 groups are comparable in the bacterial composition at the phylum (**A**) and family levels (**B**). Microbial diversity is not significantly different between the placebo and the ID-JPL934 groups (**C**, *p* > 0.05). In β-diversity index, no significant difference was observed between the two groups (PERMANOVA *p* > 0.05, Bray–Curtis distance, **D**). The figure shows the ordination plot of the OTUs at the phylum level (**E**). Scatter plots of first 2 PC loadings in the canonical analysis of principal coordinates (**F**,**G**). Arrows indicate components of the clinical variables. Some of the clinical variables including stool consistency and fiber intake appear to correlate with the ordination. In contrast, age, BMI, and abdominal symptom show limited correlation with the ordination. CHO, carbohydrate; BMI, body mass index.

**Figure 3:**
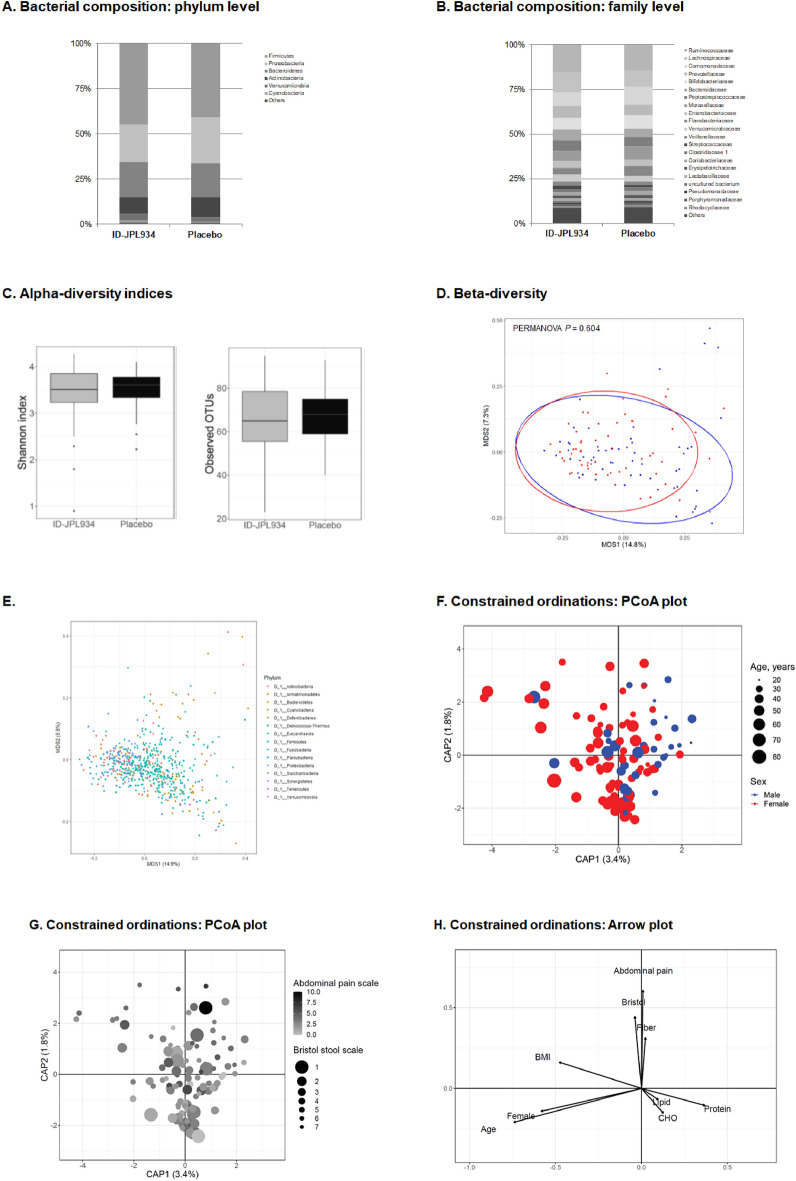
Summary of metagenomic analysis of the microbiome originating from bacteria-derived extracellular vesicles at baseline. The placebo group and the ID-JPL934 group are comparable in bacterial composition at the phylum (**A**) and family levels (**B**). Microbial diversity did not differ between the placebo group and the ID-JPL934 group (**C**, *p* > 0.05). No significant difference in β-diversity index was observed between the two groups (PERMANOVA *p* > 0.05, Bray–Curtis distance, **D**). The figure represents the ordination plot of the OTUs at the phylum level (**E**). Scatter plots of first 2 PC loadings in the canonical analysis of principal coordinates (**F**–**H**). Arrows indicate components of clinical variables. Some of the clinical variables including stool consistency, sex, age, abdominal pain, and fiber intake appear to correlate with the ordination. CHO, carbohydrate; BMI, body mass index.

No differences in microbial diversity (α-diversity) or bacterial composition (β-diversity) were detected between the two groups at baseline both in bacteria-derived EVs and bacterial cells (Shannon index *p* > 0.05 and PERMANOVA *p* > 0.05, Figs. 2A–E and 3A–E).

We then evaluated the possible association between clinical variables and gut microbiome. Interestingly, under canonical analysis of principal coordinates (CAP)-constrained ordinations the microbiome from fecal bacterial cells was substantially affected by stool consistency (Bristol stool scale) but not by age (Fig. 2F,G). In contrast, age as well as stool consistency affected the microbiome form bacteria-derived EVs (Fig. 3F–H). In addition, sex, BMI, abdominal pain, and dietary factors (carbohydrate, fat, protein, and dietary fiber intake) also influenced microbiome originating from bacteria-derived EVs or bacterial cells.

**Figure 4:**
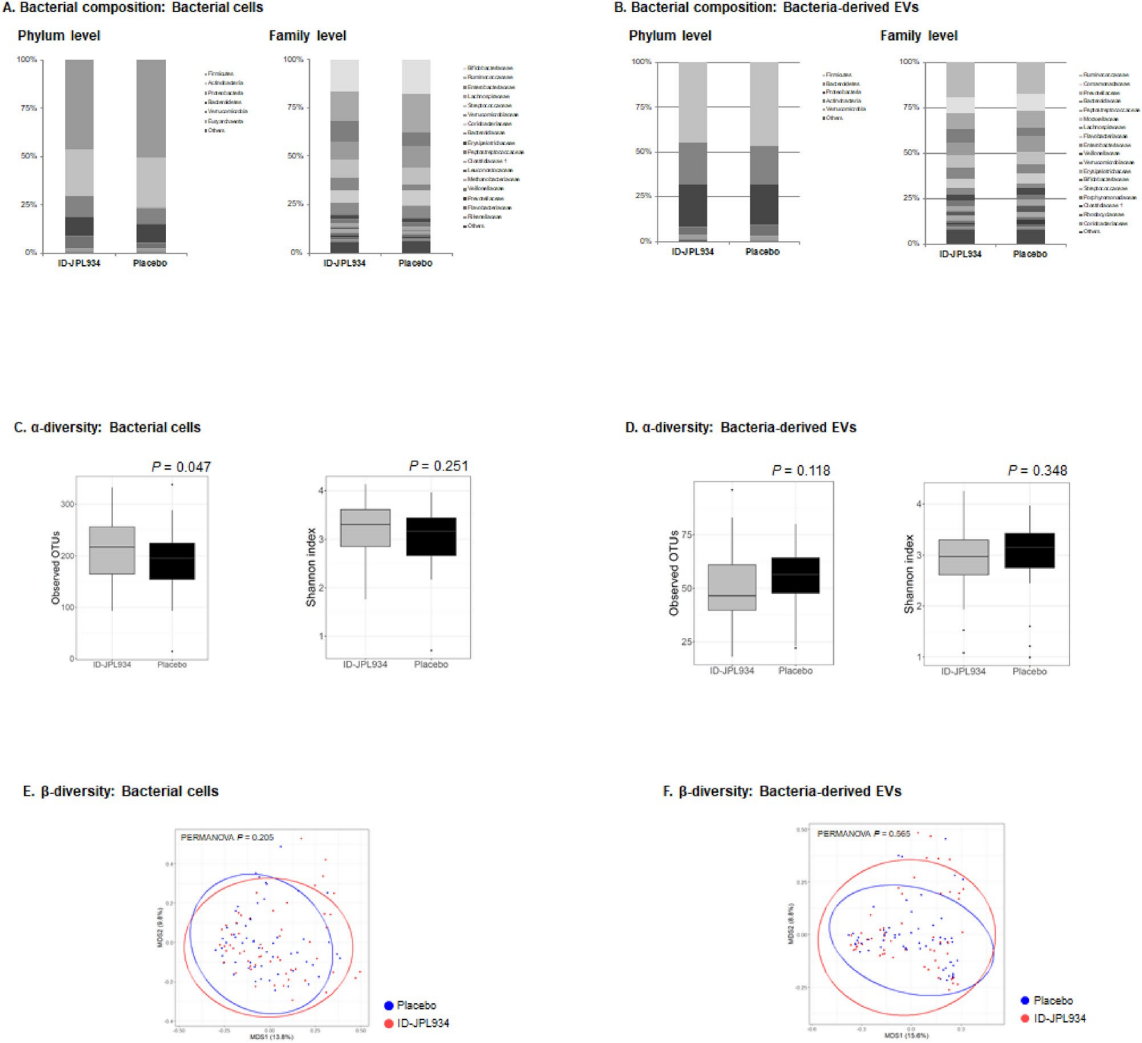
Summary of metagenomic analyses of the ID-JPL934 group and the placebo group at week 8. The compositions of bacterial cells (**A**) and bacteria-derived EVs (**B**) in feces of the ID-JPL934 group and the placebo group at 8 weeks is comparable. The microbial diversity of bacterial cells was decreased in ID-JPL93 group compared with the placebo group based on observed OTU counts (*p* = 0.047) but not according to Shannon index (**C**). The microbial diversity of bacteria-derived EVs was not different between the two groups (**D**). In bacterial cells (**E**) and bacteria-derived EVs (**F**), no significant differences in β-diversity index were observed between the two groups (PERMANOVA *p* > 0.05, Bray–Curtis distance).

Next, we compared the fecal microbiome profile of the ID-JPL934 group with that of the placebo group at 8 weeks. The observed OTU count of the microbiome from bacterial cells was increased in the ID-JPL934 group compared with the placebo group (*p* = 0.047 by Student’s t-test, Fig. 4C). However, the bacterial cell composition did not differ between the two groups (PERMANOVA *p*-value = 0.205, Fig. 4A,E). The results of microbiome analysis of bacteria-derived EVs in feces according to microbial diversity and bacterial composition were insignificant (Fig. 4B,D,F). Although the relative abundances of taxa including Lachnospiraceae, Ruminococcaceae, *Dorea*, and *Faecalibacterium* in non-EV bacterial microbiome and *Bifidobacterium*, *Haemophilus,* and *Akkermansia* in bacteria-derived EV microbiome were found to be different between the two groups at week 8 (nominal *p*-value < 0.05), they were not significant after multiple comparison correction (FDR q-value > 0.05, Supplementary Tables S2 and S3).

### Analysis of quantitative changes in probiotic levels of feces from baseline to week 8 via quantitative polymerase chain reaction

**Figure 5:**
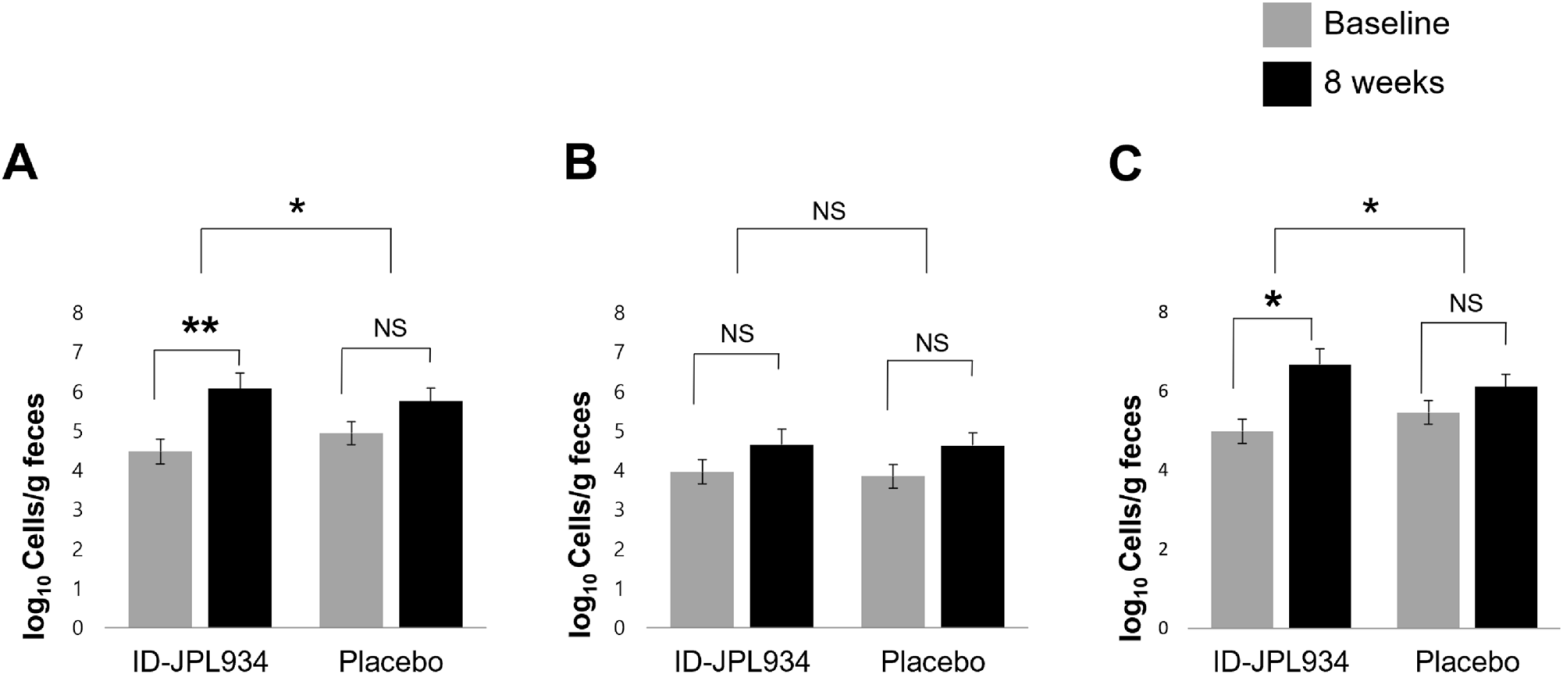
Quantitative changes in fecal probiotic levels at baseline and at week 8. (**A**) *Lactobacillus johnsonii* IDCC9203, (**B**) *Lactobacillus plantarum* IDCC3501, (**C**) *Bifidobacterium lactis* IDCC4301; *P*-values within the group were calculated using paired t-test; *P*-values between the 2 groups were calculated using repeated-measures analysis of variance. Means and standardized error are shown. *P < 0.05, **P < 0.001. NS, not significant.

Higher levels of species-specific sequences associated with probiotic formulations were detected in the fecal DNAs of subjects treated with ID-JPL934 than in those in the placebo group, based on quantitative polymerase chain reaction (qPCR) results. Specifically, the levels of *Lb. johnsonii* and *B. lactis* were significantly increased in the ID-JPL934 group (*p* < 0.05 by repeated measure analysis of variance) as shown in Fig. 5A,C, respectively, but not those of *Lb. plantarum* (Fig. 5B).

### Changes in serum inflammatory cytokine levels from baseline to week 8

The levels of serum IL-6 were significantly decreased in the ID-JPL934 group after the treatment (*p* < 0.001 by paired t-test), while no significant changes were found in the levels of the placebo group (*p* = 0.306), and the difference was significant (*p* = 0.016 by repeated measure analysis of variance, Supplementary Figure S2A). In contrast, the levels of serum TNF-α were significantly increased in the placebo (*p* = 0.018), but remained unchanged in the ID-JPL934 group (*p* = 0.190). The changes were not significantly different between the 2 groups (*p* = 0.673, Supplementary Figure S2B).

## Discussion

In this randomized, double-blinded, placebo-controlled trial, 112 individuals with abnormal bowel movement symptoms were enrolled, and 104 of the participants completed the study protocol. The study included only patients manifesting abnormal bowel movement symptoms without meeting the IBS criteria stipulated by validated ROME III questionnaire because the study was supported by the Korean government, which required evaluation of the efficacy of probiotics in a healthy population. Thus, we assessed the efficacy and efficiency of daily oral administration of probiotic combination ID-JPL934 for 8 weeks in reducing abnormal bowel movement symptoms in individuals exhibiting mild abdominal symptoms.

Study participants were randomized to receive a daily dose of either ID-JPL934 or placebo for 8 weeks. The baseline characteristics of subjects from the two groups were compared after randomization. Parameters including gender, age, body mass index, and lower abdominal symptoms appeared to be similar, except for flatulence (Table 1). That is, subjects in the ID-JPL934 group scored significantly lower on flatulence than those in the placebo group at baseline. During the study period, however, the two groups showed similar reduction in flatulence score (Supplementary Fig. S1), so changes in flatulence score did not differ between the two groups (*p* > 0.05, Table 2). Therefore, the difference in flatulence score at baseline may not substantially affect the primary outcome.

Our data demonstrated that ID-JPL934 administration during the study period was associated with a decrease in a few abnormal bowel movement symptoms without triggering any severe adverse effects (Fig. 1 and Table 2). This finding suggests that the probiotic preparation of ID-JPL934 is both safe and effective in reducing minor lower abdominal symptoms in the healthy Korean population. In fact, the efficacy of probiotics has been previously reported for various indications^10^. Treatment with specific probiotics are beneficial not only for healthy individuals with certain lower GI symptoms, but also for patients manifesting IBS, antibiotic-associated diarrhea, and diarrhea following *Helicobacter pylori* eradication therapy^11–14^. According to a recent meta-analysis, the specific probiotics may help reduce abdominal pain, bloating/distension, and constipation, but not diarrheal symptoms in patients with IBS^15^. In the present study, supplementation with multiple probiotic strains in the form of ID-JPL934 was effective in alleviating bloating and abdominal pain (Table 2).

One of the study goals was to determine the impact of probiotic intake on overall changes in intestinal microbial flora via metagenomic analyses. A recent systematic review of pertinent literature has demonstrated a lack of evidence of the impact of probiotics on fecal microbiota composition in healthy adults^16^. However, fecal microbiome contains not only the microbiome in bacteria-derived EVs but also the bacterial microbiome. In fact, bacteria-derived EVs might be closely related to some diseases^17^. In addition, bacteria-derived EVs may play a role in the treatment of diseases by either promoting or inhibiting host immunity^18–20^. A recent study has shown that *Akkermansia muciniphila*-derived or *Lb. plantarum-*derived EVs exhibit anti-inflammatory activity^21,22^. However, no studies investigated whether probiotic supplementation can change the compositions of gut bacteria-derived EVs. Against this background, we isolated EVs in feces, and analyzed the microbiome from bacteria-derived EVs distinct from microbiome from non-EV bacterial cells. Gut microbiome is affected by age, sex, BMI, dietary intake, and stool consistency^23,24^. In the study population, non-EV bacterial microbiome was affected by stool consistency and probably altered by dietary fiber but was not affected by sex, obesity, or abdominal pain (Fig. 2). In contrast, the microbiome from bacteria-derived EVs was more affected by age, sex, stool consistency, BMI, and abdominal pain (Fig. 3). Our findings suggest the distinct roles of bacteria and bacteria-derived EVs in health and disease^25^.

Unfortunately, the microbial diversity and compositions of bacterial cells and bacteria-derived EVs did not differ significantly between the ID-JPL934 group and the placebo group at week 8 (Fig. 4). When the two groups were compared at week 8, no taxon showed a significant difference (FDR q < 0.05) in relative abundance (Supplementary Tables S2 and S3). The findings suggest that probiotic supplementation does not result in substantial changes in gut microbiota profiles. The composition of fecal microbiome between baseline and at week 8 in the groups treated with ID-JPL934 or placebo revealed no significant differences in microbiome originating from bacterial cells (Supplementary Figure S3 and Supplementary Tables S4 and S5). In case of microbiome from bacteria-derived EVs, however, the microbial diversity was significantly decreased, and the bacterial composition was significantly different at week 8 compared with baseline in the placebo group as well as the ID-JPL934 group (Supplementary Figure S4 and Supplementary Tables S6 and S7). Interpretation of these results is extremely limited. First, the profiles of bacteria-derived EVs might be more affected by diet^25,26^, suggesting possible changes in dietary habits during the study period in both the test and placebo groups. However, quantitative evaluation of dietary intakes based on 72-h recall method revealed no significant differences in dietary intake between baseline and week 8 in each group (Supplementary Table S1). One possible explanation was that the placebo group also showed an improvement in lower GI symptoms (placebo effect, Table 2). Abdominal symptoms might be more related to bacteria-derived EVs than to bacteria per se*.* According to the brain-gut-microbiome axis, the improvement in lower abdominal symptoms might be associated with changes in gut microbiota, particularly bacteria-derived EVs^27–29^. However, no studies have explored functional GI symptoms related to bacteria-derived EVs, which makes it difficult to draw any definitive conclusions in this study, suggesting the need for additional studies.

This study also showed that fecal probiotic formulations were detected more in the ID-JPL934 group than in the placebo group (Fig. 5). This finding suggests that the probability of improvement in patient symptoms might be related to the strain ingested. However, because it is quantitatively measured by qPCR, it is difficult to demonstrate its actual function in the intestine. Nevertheless, treatment with ID-JPL934 yielded favorable outcomes in terms of inflammatory cytokine levels such as IL-6 in the blood (Supplementary Figure S2). All three strains used in this study exhibited anti-inflammatory and antioxidant effect through animal experiments and in vitro studies^9^. The three strains used in ID-JPL934 might have synergistic effects with each other. However, the changes in gut microbial abundance of *Lb. Johnsonii* and *B. lactis* were not confirmed via 16S rDNA analysis, warranting the need for further experimental and clinical studies.

This study has the following limitations. First, it was not a study involving IBS patients. Thus, further studies are needed to evaluate the efficacy and safety of probiotic supplementation in patients diagnosed with IBS. Second, the sample size was insufficient to perform a metagenomic analysis adjusting the results for diet. Third, although quantitative changes in fecal probiotic levels were confirmed via qPCR, it was unclear whether the probiotics were successfully colonized in the gut. Furthermore, a qualitative analysis of their mode of action in the intestine could not be conducted. Fourth, the questionnaire for abnormal bowel movement symptoms was not validated in Korean. To date, no validated IBS symptom severity questionnaire in the Korean language is available. The questionnaire used in this study was a modified version gastrointestinal symptom rating scale or GSRS^30^. However, the primary outcome was overall abdominal symptom improvement which was evaluated using a separate questionnaire. Fifth, all study subjects were recommended their usual diet and lifestyle throughout the entire study period and prohibited from using probiotics or medications that might affect bowel symptoms. No significant differences in diet were detected during the study period (Supplementary Table S1). However, we could not determine whether or not other lifestyle factors such as exercise or sleep were changed during the study period. Lifestyle changes may have affected abdominal symptoms and/or gut microbiome profiles. However, any changes in lifestyle during the study period were not different between the two groups. In contrast, the probiotics administered might affect mood or sleep pattern, and thereby affect abdominal symptoms.

Despite the foregoing limitations, the study results were encouraging in demonstrating the efficacy of probiotic supplementation in ameliorating lower abdominal symptoms in healthy individuals with mild GI disturbances. In addition, it was the first study of changes associated with fecal bacteria-derived EVs after probiotic supplementation.

In conclusion, ID-JPL934 may be effective in alleviating abnormal abdominal movements. While ID-JPL934 treatment may not affect the overall gut microbial composition, the treatment may increase the gut microbial abundance of *Lactobacillus johnsonii* and *Bifidobacterium lactis*.

## Methods

### Subjects and study design

A total of 117 individuals aged 18–80 years with abnormal bowel movement symptoms who understood the study content and agreed to participate in this clinical trial were enrolled. In this study, only individuals who manifested abdominal pain or discomfort which did not meet the IBS criteria based on validated Korean ROME III questionnaire during the screening period were included^31^. In addition, individuals were excluded if they met any of the following exclusion criteria: (1) lactose intolerance; (2) severe systemic illnesses; (3) a history of any cancer; (4) a history of psychiatric disorder; (5) exposure to psychiatric drugs, antibiotics, and steroids within 3 months, (6) intake of probiotic supplements within 2 weeks; and (7) a history of any abdominal surgery except for appendectomy and hernia.

A total of 112 subjects fulfilled the inclusion criteria without meeting the exclusion criteria. The eligible subjects were randomized to either the ID-JPL934 group or the placebo group in blocks of 4 using a computer-generated table. All subjects and investigators, except for the study coordinator, were blinded to the randomization process until study completion. Participants received ID-JPL934 capsule (containing 1.0 × 10^10^ colony-forming units of three live bacterial strains combined; *Lb. johnsonii* IDCC 9203, *Lb. plantarum* IDCC 3501, and *B. lactis* IDCC 4301 at a 1:1:1 ratio) or equivalent vehicle for 8 weeks.

Subjects were provided with the investigational products when they visited the clinic and underwent assessment for compliance, symptoms, and safety at 2, 6, and 8 weeks after the first administration. Subjects visited the clinic to obtain their investigational products and to undergo assessments for compliance, symptoms, and safety at 2, 6, and 8 weeks after the first administration. At every visit (weeks 0, 2, 6, and 8), participants were asked to complete the stool form (Bristol stool form scale, BSFS), average number of spontaneous complete bowel movements per day, and the 10-point visual analogue scale (VAS) of lower gastrointestinal symptoms associated with abnormal bowel movements (abdominal pain, abdominal discomfort, constipation, diarrhea, bloating, and flatulence) during the past week. At 8 weeks, the general symptom relief was evaluated using a 10-point VAS questionnaire using items such as “Thinking about your lower abdominal symptoms such as abdominal pain, abdominal discomfort, and diarrhea or constipation during the past week, how much do you think your overall abdominal symptoms have improved after taking the dietary supplement?” During the study period, none of the study participants were allowed to use antispasmodic agents (i.e., cimetropium, mebevarine, pinaverium, and trimebutine), prokinetics (i.e., domperidone, levosurpiride, itopride, and mosapride), antidepressants (i.e., tricyclic antidepressants and selective serotonin reuptake inhibitors), anti-diarrheal drugs such as loperamide, laxatives, antibiotics or probiotics. Also, subjects were educated to follow their usual diet and lifestyle. We also collected follow-up fecal samples (Supplementary Figure S5). All study participants were required to complete the Bristol stool scale questionnaire at baseline and at 2, 6, 8 weeks after treatment. Also, a structured 3-day recall questionnaire was administered at baseline and at 8 weeks. Good compliance was defined by 80 to 120% adherence of the allocated treatment.

During the study period, two participants in the ID-JPL34 group and six subjects in the placebo group dropped out (Supplementary Figure S6), including five subjects who failed to meet the criteria for compliance and three who did not visit the research center as scheduled. This study analyzed data via both intention-to-treat analysis (primary outcome) and per-protocol analysis (primary and secondary outcomes).

### Institutional review board

All patients provided informed consent. This study was approved by the Institutional Review Board of Seoul National University Bundang Hospital (accession number: B-1702/384-002). Informed written consent was obtained from the subjects after the investigators provided detailed explanation for about 15 min. All methods were carried out in accordance with relevant guidelines and regulations. This study was retrospectively registered at ClinicalTrials.gov (NCT03395626, date of first registration: 10/01/2018).

### Primary outcome and secondary outcomes

The primary outcome of this study was improvement in overall abdominal symptoms at week 8. Secondary outcomes were (1) changes in stool form, stool frequency, and the abnormal bowel movement symptoms; (2) quantitative changes in fecal probiotic levels following treatment (*Lb. johnsonii*, *Lb. plantarum,* and *B. lactis*); and (3) changes in fecal microbiome profiles, especially microbiome originating from bacterial cells and bacteria-derived extracellular vesicles (EVs) (see below).

### Immunological markers

To investigate the changes in serum cytokine levels (IL-6 and TNF-α) during the study period, samples were collected by centrifugation (10,000×*g*, 15 min) after 2 h of blood collection, and stored at − 80 °C until examination. Enzyme-linked immunosorbent assay (ELISA) was performed according to the manufacturer’s protocol (R&D Systems, USA). The sensitivity and assay limits of Human TNF-alpha Quantikine ELISA Kit (https://www.rndsystems.com/products/human-tnf-alpha-quantikine-elisa-it_dta00d) were 6.23 pg/mL and 15.6–1000 pg/mL, respectively. The sensitivity and assay limits of Human IL-6 Quantikine ELISA Kit (https://www.rndsystems.com/products/human-il-6-quantikine-elisa-kit_d6050) were 0.7 pg/mL and 3.1–300 pg/mL, respectively.

### Fecal samples

The study subjects provided fecal specimens at baseline and week 8. As soon as the specimens were taken, they were collected into sterile containers, brought to the laboratory the same day in a frozen condition, and stored at − 80 °C until analysis. For qPCR analysis, genomic DNA samples were extracted from the entire fecal sample. In contrast, metagenomic analysis was performed using Illumina MiSeq platform targeting the 16S rDNA after separating EVs from feces (see below)^32^.

### Preparation of genomic DNAs from reference strains and fecal samples

Real-time qPCR was conducted with bacterial genomic DNAs obtained from pure cultures or fecal samples using the QIAamp DNA Stool Mini Kit (Qiagen, Germany). Genomic DNA was extracted from 1 mL of pure culture according to the manufacturer’s instructions.

### Real-time quantitative PCR

The metagenomic analysis including separating EVs from feces was performed as reported previously^33,34^. Real-time qPCR was carried out using a CFX Real-Time PCR (Bio-Rad, IL, USA). Supplementary Table S8 lists species-specific primers for PCR. All primers were synthesized by Bioneer, Korea. The primer specificity was previously verified using DNAs derived from closely or distantly-related bacteria. Quantitative PCR was performed in 96-well plates with a final volume of 20 μL consisting of 1 μL of fecal DNA, 0.5 μL of primers (10 pmol each), 10 μL SYBR Green I master (Roche, Germany), and 8 μL of H_2_O. The PCR amplification program consisted of a pre-incubation step at 94 °C for 4 min followed by 55 cycles of amplification step (denaturation at 94 °C for 15 s, primer annealing at 55 °C for 15 s, and elongation at 72 °C for 20 s). Melting curves were obtained by heating samples from 50 to 90 °C at the rate of 5 °C/s.

### Isolation of extracellular vesicles in feces and DNA extraction

EVs in human feces were isolated via centrifugation, as reported previously^34,35^. Human stool samples were filtered via a cell strainer after incubation in 10 mL of phosphate-buffered saline for 24 h. To separate EVs from stool samples, EVs in the samples were isolated via centrifugation at 10,000×*g* for 10 min at 4 °C to obtain the pellet of stool samples containing bacterial cells, while the supernatant of stool samples contained EVs. Bacteria and foreign particles were eliminated from the sample supernatants via sterilization using a 0.22 μm filter. To extract DNAs from bacterial cells and bacterial EVs, bacteria and EVs were boiled for 40 min at 100 °C. To eliminate the residual floating particles and debris, the supernatant was collected after centrifugation at 13,000 rpm for 30 min at 4 °C. DNA was extracted using a PowerSoil DNA Isolation Kit (MO BIO, Carlsbad, CA, USA), following the standard manufacturer’s protocol. The DNA extracted from the bacterial cells and EVs in each sample was quantified using a QIAxpert system (QIAGEN, Hilden, Germany). In this study, we analyzed the DNAs from bacterial cells (microbiome originating from bacterial cells) and DNAs from bacterial EVs (microbiome originating from bacteria-derived EVs) separately.

### Bacterial metagenomic analysis of human stool samples

Microbiome originating from bacteria-derived EVs and microbiome originating from bacterial cells was amplified respectively with 16S_V3_F (5′-TCGTCGGCAGCGTCAGATGTGTATAAGAGACAGCCTACGGGNGGCWGCAG-3′) and 16S_V4_R (5′-GTCTCGTGGGCTCGGAGATGTGTATAAGAGACAGGACTACHVGGGTATCTAATCC-3′) primers specific for V3-V4 hypervariable regions of the 16S rDNA gene^33^. Libraries were prepared using PCR products based on MiSeq System guide (Illumina, San Diego, CA, USA) and quantified using QIAxpert (QIAGEN, Hilden, Germany). Each amplicon was then quantified, set at an equimolar ratio, pooled, and sequenced with MiSeq (Illumina, San Diego, CA, USA), according to the manufacturer’s recommendations.

### Analysis of the fecal microbiota

Paired-end reads that matched adapter sequences were trimmed with cutadapt version 1.1.6^36^. The resulting FASTQ files containing paired-end reads were merged with CASPER and then quality filtered with Phred (Q) score-based criteria described by Bokulich^37,38^. Any reads shorter than 350 bp or longer than 550 bp after merging were discarded. To identify chimeric sequences, a reference-based chimera detection step was performed with VSEARCH against SILVA gold database^39,40^. Next, sequence reads were clustered into Operational Taxonomic Units (OTUs) using VSEARCH with de novo clustering algorithm under a threshold of 97% sequence similarity. Representative OTU sequences were finally classified using SILVA 128 database with UCLUST (*parallel_assign_taxonomy_uclust.py* script on QIIME version 1.9.1) under default parameters^41^. In this study, rare OTUs with mean relative abundances < 0.1% were discarded, and the remaining OTU counts converted to log_10_ transformed relative abundances (with the addition of 10^−6^ for zero counts). The OTU counts were collapsed by shared taxonomy at all taxonomic levels from phylum to genus using ‘tax_glom’ function in the ‘phyloseq’ package using R^42^.

### Statistical analysis

For sample size calculation, the difference in overall symptom improvement at week 8 between the two groups was assumed at 20%, and the estimated standardized deviation was 30%. At a statistical power of 0.80 with a two-sided significance level of 0.05, the number of subjects in each group was calculated as 47. Considering a dropout rate of 20%, the sample size was determined as 112 (56 in each group).

Baseline demographics and clinical data are reported for all subjects as *n* (percentage) and mean with standard deviation (SD). Food intake data based on the 3-day recall questionnaires were analyzed via the Computer Aided Nutritional Analysis version 3.0 (CAN-pro 3.0, Nutritional Assessment Program, 2006, The Korean Nutrition Society, Seoul, Korea)^38^. Relief of overall symptoms and abnormal bowel movement symptoms at 8 week were compared with their baseline values using a paired t-test. A general linear model was used to compare quantitative changes in probiotic levels of the fecal samples before and after the study period between the two groups.

For metagenomic analysis, the OTU table was rarefied to an even depth, followed by the estimation of α-diversity indices (observed species and Shannon diversity index) and testing for significant differences between the two groups using Student’s t-test. Log-transformed relative abundance in each taxon was compared between the two groups before and after treatment by Student’s t-test with false discovery rate (FDR) corrected q-values. Beta-diversity distances (Bray–Curtis distances) were calculated using rarefied OTU tables. For α- and β-diversity analyses, either QIIME or ‘phyloseq’ package in R was used^43^. Permutational multivariate analysis of variance (PERMANOVA) and homogeneity of dispersion tests were performed using the ‘adonis’ function of the ‘vegan’ package in R^44^.

IBM SPSS Statistics v.22.0 for Windows (IBM Corp., Armonk, NY, USA) and R statistical software (Foundation for Statistical Computing, Vienna, Austria) programs were used for all statistical analyses. A *p*-value < 0.05 was considered significant.

## Supplementary Information

Supplementary Information 1.

Supplementary Information 2.

Supplementary Information 3.

## Data Availability

The raw reads were deposited in the NCBI Sequence Read Archive (SRA) database (Accession Numbers: SRR 12072450–12072561).
