# Supplementary material for: Validity and safety of ID-JPL934 in lower gastrointestinal symptom improvement
Source: Sci Rep. 2021 Jun 22;11:13046. doi: 10.1038/s41598-021-92007-3 (PMC8219743; doi:10.1038/s41598-021-92007-3)
Supplement: Supplementary file 1 — Supplementary Information 1. [file 41598_2021_92007_MOESM1_ESM.pdf]

Validity and Safety of ID-JPL934 in Lower Gastrointestinal Symptom Improvement

Cheol Min Shin[1], Yoon Jin Choi[1], Dong Ho Lee[1], Jin Seok Moon[2], Tae-Yoon Kim[2], Yoon-Keun Kim[3], Won-Hee Lee[3], Hyuk Yoon[1], Young Soo Park[1] & Nayoung Kim[1]

[1]Department of Internal Medicine, Seoul National University Bundang Hospital, Seongnam, Gyeonggi, South Korea; [2]Research Laboratories, ILDONG pharmaceutical Co., Ltd., Hwaseong, South Korea; [3]MD Healthcare Inc., Seoul, Republic of Korea.

Supplementary Figure Legends

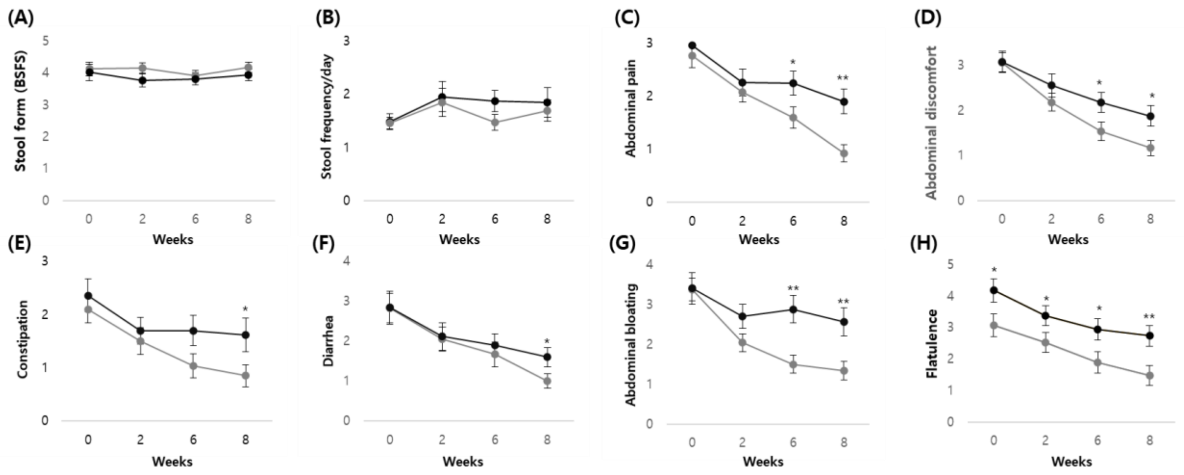

Fig S1. Changes of 10-point VAS score in stool form and the 7 abnormal bowel movement symptoms during the study period (week 0, 2, 6, 8). ID-JPL934 (black) and Placebo (gray). (A) Stool form (BSFS), (B) Stool frequency/day, (C) Abdominal pain, (D) Abdominal discomfort, (E) Constipation score, (F) Diarrhea score, (G) Abdominal bloating, (H) Flatulence; Means and SE are shown. Statistically significant differences between groups are symbolized by *($p < 0.05$) and **($p < 0.01$). VAS, visual analogue scale; BSFS, Bristol stool form scale.

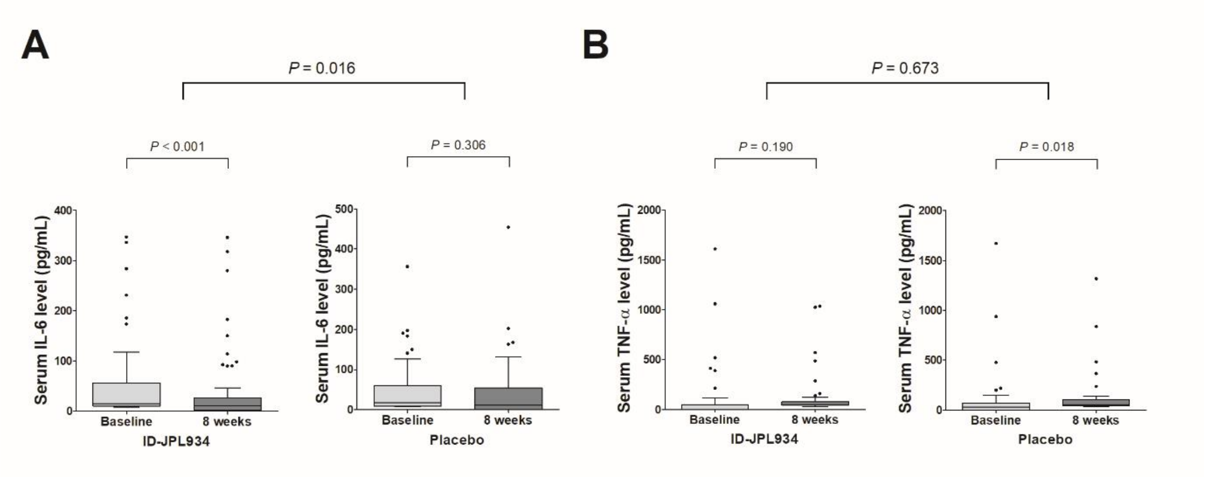

Fig S2. Effects of ID-JPL934 on blood levels of (A) IL-6 and (B) TNF- α at each time point (baseline and week 8, N = 104). *P*-values within the group were calculated by paired t-test. *P*-values between the 2 groups were calculated by repeated measure analysis of variance. Median and the interquartile range were shown.

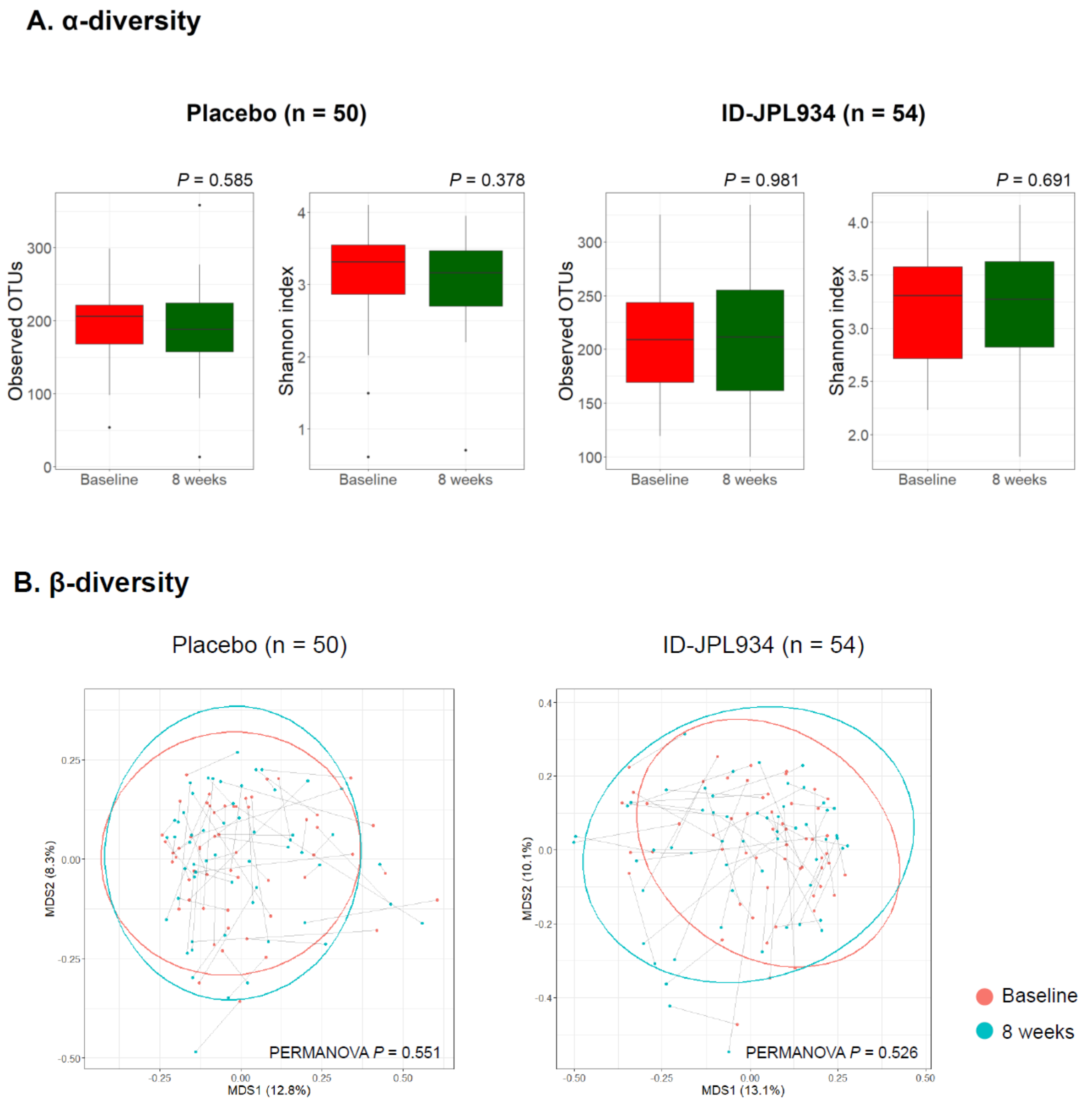

Fig S3. Summary of the metagenomic analysis of the microbiome originating from bacterial cells between baseline and week 8. There were no significant differences observed in terms of microbial diversity (**A**) and beta-diversity (Bray-Curtis distance, **B**) before and after treatment in both groups (all $p > 0.05$)

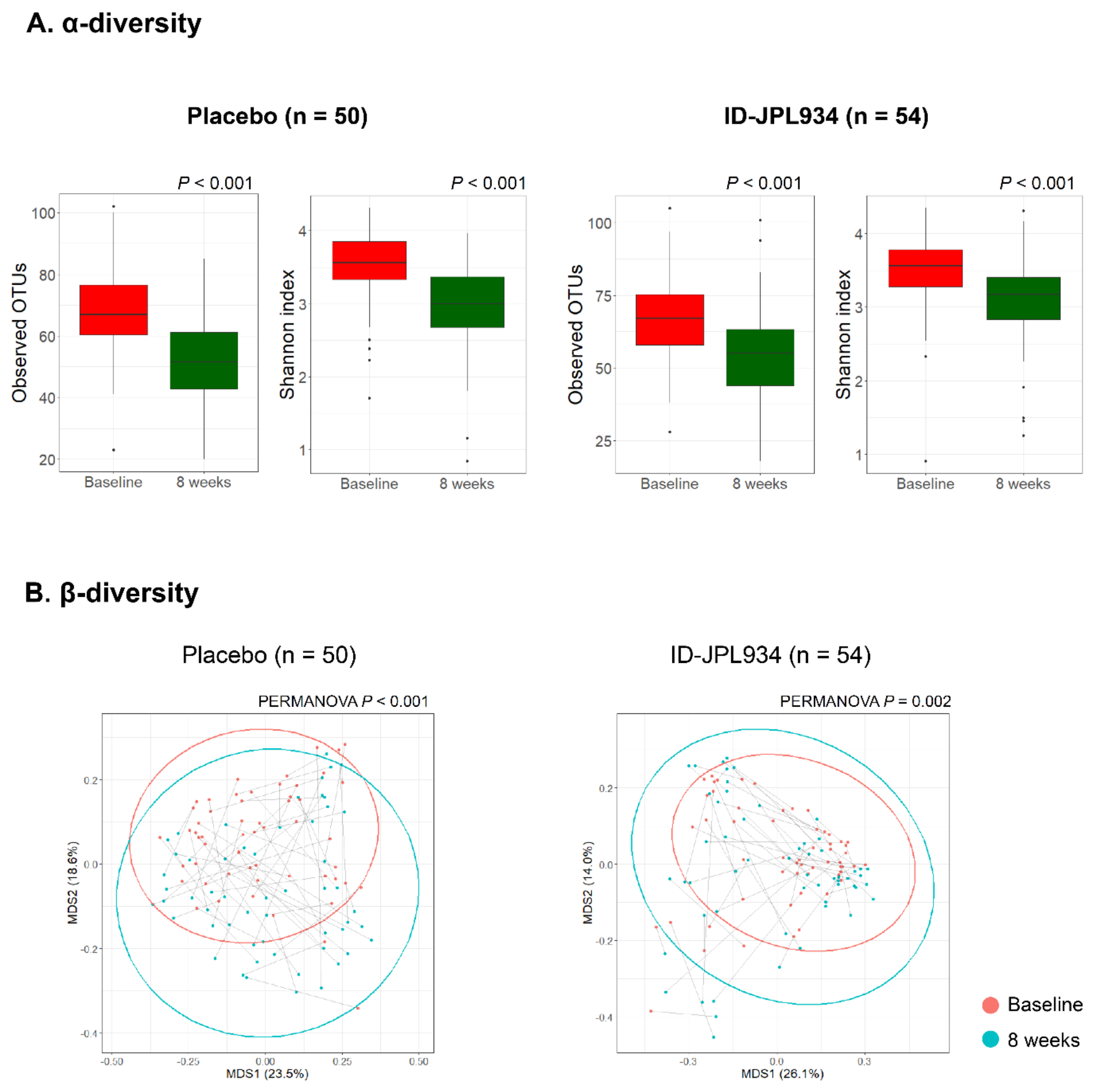

Fig S4. Summary of the metagenomic analysis of the microbiome originating from bacteria-derived extracellular vesicles between baseline and week 8. There was a significant decrease of microbial diversity in the ID-JPL934 group as well as the placebo group (p < 0.001 by Student's t-test, **A**). A significant difference was observed in beta-diversity analysis before and after treatment in both groups (PERMANOVA p < 0.05, Bray-Curtis distance, **B**).

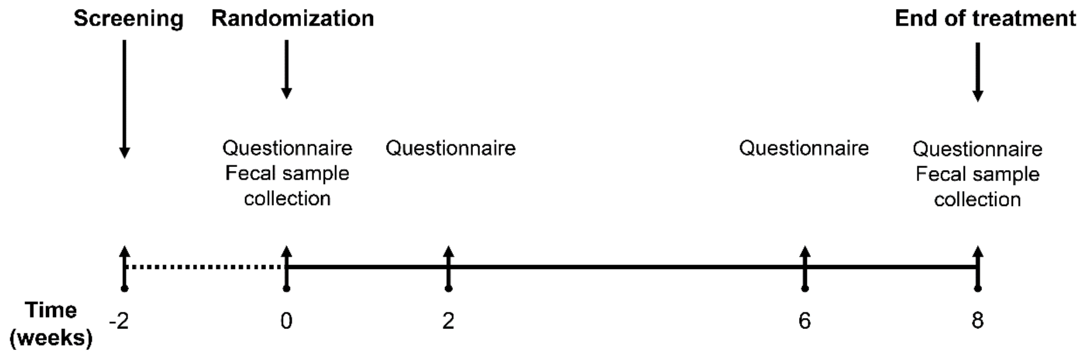

Fig S5. Study protocol

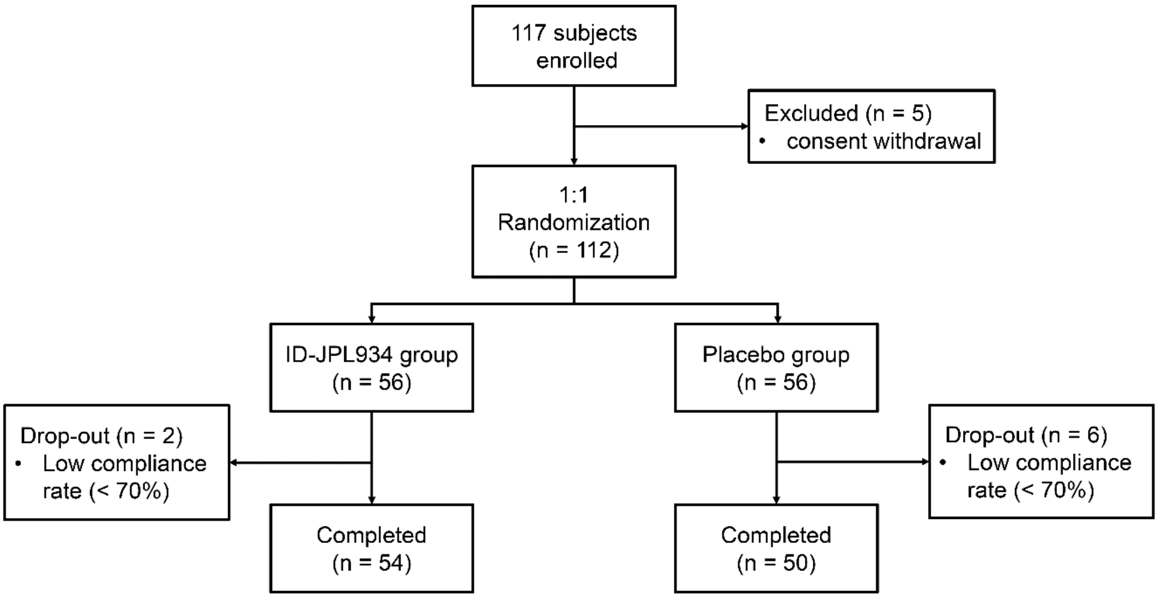

Fig S6. Flow chart of the study
