# Supplementary material for: Validity and safety of ID-JPL934 in lower gastrointestinal symptom improvement
Source: Sci Rep. 2021 Jun 22;11:13046. doi: 10.1038/s41598-021-92007-3 (PMC8219743; doi:10.1038/s41598-021-92007-3)
Supplement: Supplementary file 2 — Supplementary Information 2. [file 41598_2021_92007_MOESM2_ESM.pdf]

Validity and Safety of ID-JPL934 in Lower Gastrointestinal Symptom Improvement

Cheol Min Shin[1], Yoon Jin Choi[1], Dong Ho Lee[1], Jin Seok Moon[2], Tae-Yoon Kim[2], Yoon-Keun Kim[3], Won-Hee Lee[3], Hyuk Yoon[1], Young Soo Park[1] & Nayoung Kim[1]

[1]Department of Internal Medicine, Seoul National University Bundang Hospital, Seongnam, Gyeonggi, South Korea; [2]Research Laboratories, ILDONG pharmaceutical Co., Ltd., Hwaseong, South Korea; [3]MD Healthcare Inc., Seoul, Republic of Korea.

Supplementary Tables (S1-S8)

Table S1. Dietary intake of the study participants during the 8 weeks intervention period

Table S2. Summary of taxa of which relative abundances were different between the placebo group and ID-JPL934 administration group at week 8 (microbiome originating from bacterial cells)

Table S3. Summary of taxa of which relative abundances were different between the placebo group and ID-JPL934 administration group at week 8 (microbiome originating from bacteria-derived extracellular vesicles)

Table S4. Summary of taxa of which relative abundances at week 8 were significantly different from those at baseline in the placebo group (microbiome originating from bacterial cells)

Table S5. Summary of taxa of which relative abundances at week 8 were significantly different from those at baseline in the ID-JPL934 group (microbiome originating from bacterial cells)

Table S6. Summary of taxa of which relative abundances at week 8 were significantly different from those at baseline in the placebo group (microbiome originating from bacteria-derived extracellular vesicles)

Table S7. Summary of taxa of which relative abundances at week 8 were significantly different from those at baseline in the ID-JPL934 group (microbiome originating from bacteria-derived extracellular vesicles)

Table S8. Oligonucleotides used for quantitative real-time PCR assays
